# Feeding practices in 6–24-month-old children with chronic cholestatic liver diseases: a mixed-method study

**DOI:** 10.1186/s12887-020-02290-8

**Published:** 2020-08-24

**Authors:** Xiao Chen, Jianshe Wang, Yi Lu, Xinbao Xie, Ying Gu, Jos M. Latour, Yuxia Zhang

**Affiliations:** 1 Department of Liver Disease, Children’s Hospital of Fudan University, Shanghai, People’s Republic of China; 2 Department of Nursing, Zhongshan Hospital of Fudan University, No.180, Fenglin road, Shanghai, People’s Republic of China; 3 Department of Nursing, Children’s Hospital of Fudan University, Shanghai, People’s Republic of China; 4 School of Nursing and Midwifery, Faculty of Health and Human Sciences, University of Plymouth, Plymouth, UK

**Keywords:** Cholestatic liver disease, Feeding practices, Medium-chain triglyceride, Complementary food, Feeding experiences

## Abstract

**Background:**

Children with chronic cholestatic liver diseases have a high risk of malnutrition. However, nutritional management in China has received little attention, and there has been limited evidence regarding improving these practices. This study aimed to evaluate the feeding status of chronic cholestatic children aged 6–24 months and to explore their parents’ experiences with feeding practices.

**Methods:**

A mixed-method study was conducted among parents of 74 children with chronic cholestatic liver diseases. The Infant and Young Child Feeding Index (ICFI) was used to collect quantitative data of feeding practices. In-depth interviews with parents were performed to generate qualitative data. Multivariate analysis was conducted to identify predictors of inappropriate feeding practices. Qualitative data were analyzed using thematic analysis.

**Results:**

Only 16.2% of the children had appropriate feeding practices. In terms of dairy products, the rate of breastfeeding, medium-chain triglyceride formula feeding, and regular formula feeding were 25.7, 39.2 and 44.6% respectively. The complementary feeding rate was 68.8%, and the age of adding complementary foods was 6.9 ± 1.7 months. Consumption of foods from animal sources was suboptimal, 66.7% of the children aged 6–8 months and 45.5% of the children aged 9–11 months had carbohydrates as a single source of complementary foods and had no intake of meat, eggs or bean products, while in the age group 12–24 months, 52.0% of the children had eggs less than 2 days/week, 60.0% had meat less than 2 days/week, and 60.0% had no intake of bean products. Low literacy of the primary care provider was significantly related to inappropriate feeding practices (adjusted OR 5.52; 95% CI 1.29, 23.66). The result of the interviews indicated that parents generally lacked the scientific knowledge of feeding and thought that the intake of animal source foods and oils would be a burden to the liver and cause damage to the liver functions.

**Conclusion:**

Parents generally lacked science-based feeding knowledge and the feeding practices in 6–24-month-old children diagnosed with chronic cholestatic liver diseases fell short of the recommendations in current nutritional guidelines. Appropriate interventions targeting social and cultural family circumstances should therefore be included in supporting parents in feeding practices to improve children’s clinical outcomes.

## Background

Severe malnutrition affects approximately 40–70% of the children with chronic cholestatic liver diseases [1–3]. The impacts of malnutrition on children’s outcomes include increased risks of pre- and post liver transplantation morbidity and mortality, immune suppression, and impaired neurocognitive development [4–6]. Therefore, it is important to perform nutritional management, including nutritional assessment, intervention, and follow-up, to prevent negative health outcomes among children. Nutritional assessments, the cornerstone of nutritional management, can identify the impaired nutritional status in a timely manner and can clarify the existing problems in feeding practices [7–9]. It is recommended to perform routine nutritional assessments in daily clinical practices; these assessments should be comprehensive, should involve anthropometric measurements, and detailed feeding histories, and should include social and cultural factors influencing feeding practices [9].

Only a few studies focusing on the dietary intake status of children with chronic cholestatic liver diseases have been reported. Rovner et al. investigated 26 children with Alagille syndrome and found that more than 20% of these children were consuming less than two-thirds of the recommended daily allowance (RDA) of calories, fat, calcium, vitamin D, and vitamin E [10]. Shiga et al. reported that the mean energy intake in children with biliary atresia was 84% of RDA [11].

Previous studies revealed that the feeding practices were suboptimal, but these studies lacked detailed descriptions and analyses of dietary intake and had small sample sizes. In addition, these studies were conducted before the feeding guidelines for children with chronic cholestasis were published; thus, their conclusions were drawn through comparison to the RDA of normal children instead of cholestatic children [11, 12]. Moreover, the reasons behind parents’ feeding practices remain unclear, making it difficult to cost-effectively promote behavioral changes.

Although the morbidity of children with chronic cholestatic liver diseases is low globally [13], the number of these children in China is considerable because of the large overall population. However, nutritional management in China has received little attention, and there has been limited evidence regarding improving these practices. Investigating feeding practices according to the latest guidelines for nutrition management could provide a foundation for further tailoring care plans for nutritional support.

The first 2 years after birth represent the most important period for the growth and development of a child. Nutrition during this period not only influences children’s physical development, but also profoundly affects on neurobehavioral development and healthy dietary habits [14]. For 6–24-months-old children, the nutrient requirements cannot be fully met by dairy products, and parents’ feeding behaviors play a crucial role in the nutritional management. Therefore, this study aimed to evaluate the feeding status of chronic cholestatic children aged 6–24 months and to explore their parents’ experiences with feeding practices.

## Methods

The study adopted a mixed-method design. The quantitative method used the Infant and Young Child Feeding Index survey to evaluate feeding practices. The qualitative method used semistructured interviews with parents to explore their feeding experiences and analyze barriers to appropriate feeding practices. The study is reported using the reporting guideline of ‘the quality of mixed methods studies in health services research’ [15] obtained from the EQUATOR-network website.

### Quantitative method

#### Participants

Parents of children with chronic cholestatic liver diseases were consecutively recruited from January 2016 until March 2017 at the Children’s Hospital of Fudan University in Shanghai, China. Inclusion criteria were parents who had 6–24-month-old children with chronic cholestatic liver diseases, defined as a diagnosis established by gene sequencing or presence of at least 6 months of typical clinical and biochemical derangements [16]. Parents were excluded if the child was a premature infant, had other serious diseases seriously influencing feeding, or could not receive normal feeding due to progressive deterioration of conditions, such as acute liver failure.

#### Sample size

A single population proportion formula was used to determine the sample size based on the following assumptions. The proportion of appropriate feeding practices was 80% [10], with a 95% CI, 10% margin of error (d), and 10% nonresponse rate, resulting in a sample size of 74.

#### Data collection

Children with chronic cholestatic liver diseases routinely visited the outpatient clinic for regular follow-up and adjustment of medical treatment. After visiting the outpatient service, parents of eligible children were invited to participate in the study.

Demographic characteristics, including sex, date of birth, gestational age, birth weight, literacy of parents, and monthly household income, were collected by asking the parents. Laboratory data were obtained from the medical records, including alanine aminotransferase, aspartate aminotransferase, total bilirubin, direct bilirubin, and gamma-glutamyl transpeptidase level.

Feeding practices were measured using the Infant and Young Child Feeding Index (ICFI), which was designed by the ‘Chinese Center for Disease Control and Prevention’ according to the WHO recommendations of child feeding practices [17]. The ICFI has been widely used to assess the dietary intake of children aged 6–24 months and has been suggested to be efficient in assessing parents’ feeding practice in China. Numerous studies have shown that the ICFI is strongly associated with the status of children’s growth and development [18, 19]. The ICFI is a comprehensive index and includes seven variables and thirteen items: breastfeeding, bottle feeding, dietary diversity for the past 24 h, frequency of food (grains, vegetables, fruits, eggs, flesh foods, bean products, and dairy products) for the past 7 days, first formula milk feeding age, first complementary feeding age, and frequency of complementary feeding for the past 24 h. Breastfeeding and bottle feeding were assessed by a 2-point scale (yes/no), while the other items were assessed on either a 2-point or 3-point Likert scale (Electronic Supplemental Material 1). The range of the total score was 0–23, and higher scores indicated better feeding practices. Appropriate feeding practice was determined when the score was greater than 60% of the possible score, and inappropriate feeding practice was defined as ICFI < 14. In addition, given the unique nutrient requirements of children with chronic cholestatic liver diseases [8, 20], two ad-hoc questions were added to the survey to identify the intake of dairy products and medium chain triglycerides.

#### Statistical analysis

Descriptive data were expressed as either means and SDs, medians and interquartile ranges for continuous variables or frequencies for categorical variables. The Shapiro-Wilk test showed that the levels of total bilirubin, direct bilirubin, glutamyl transpeptidase, alanine aminotransferase, and aspartate aminotransferase had nonnormal distribution. These data were therefore expressed as the median and interquartile range (IQR).

When we analyzed the determinants of feeding practice, bivariate analysis was first conducted between the feeding index and each variable, including sex of child, age of child, birth order of child, family monthly income per capita, literacy of primary care provider, presence of cholestasis (direct bilirubin), and area of residence. Primary care providers were defined as those who took the main responsibility for disease management, feeding and daily activities. The variables marginally associated (*p* < 0.10) with the feeding index in the bivariate analysis were selected for multivariate analyses. Then, multivariate analysis was conducted for the feeding index with potential risk factors. In the final model, only variables significantly associated (*p* < 0.05) with outcome variables were retained, and the adjusted odds ratio (AOR) and 95% confidence interval (CI) were reported. Children with missing data were excluded from in the model.

All statistical analyses were performed using IBM-SPSS, version 22 (IBM Corp, Armonk, NY, USA). The significance level was set at *p* < 0.05.

### Qualitative design

#### Sampling

After the survey of feeding practices, to determine the motives behind inappropriate feeding practices and promote behavioral changes, we purposefully approached participants with an ICFI score < 14. We continued data collection until thematic saturation was observed, which meant that there were no new codes or themes occurring during the ongoing analysis [21]. In total, ten mothers and one father participated in the interviews.

#### Interviews

Interviews with parents were conducted in a quiet meeting room in the hospital. A semistructured interview guide was developed. Example questions were as follows: ‘How did you feed your child?’, ‘What were the sources of your feeding knowledge?’, and ‘Can you explain any confusion or uncertainty about how to feed your child?’. The duration of the face-to-face interviews was 30–60 min. All interviews were audio-recorded and transcribed verbatim within 24 h after the interview.

#### Data analysis

Thematic analysis was carried out by structuring data, initial coding, categorizing experiences and perceptions, and identifying themes [22].

The first author (XC) read all transcripts, identified and sorted meaningful units that elucidated the study question and categorized the units into preliminary topics. Then, the interview transcripts were individually coded by two researchers (XC and YZ) and checked for similarities. Discrepancies were solved by discussion between the two researchers. Subsequently, we organized and categorized the codes into subthemes/themes, which were discussed with two other researchers (YG and JML).

### Ethical considerations

The study was approved by the Research Ethics Committee of the Children’s Hospital of Fudan University (approval number 2014–143) and was performed in accordance with the ethical standards of the 1964 Declaration of Helsinki and its later amendments. Informed consent was obtained after providing information sheet and verbally explaining the study aims and procedures. Participation was voluntary and each parent signed a written consent form.

## Results

In total, 74 children were included in the study. The demographic and clinical characteristics of the children are presented in Table 1. The etiologies of cholestasis were Alagille syndrome (*n* = 27; 36.5%), biliary atresia (*n* = 16; 21.6%), neonatal intrahepatic cholestasis caused by citrin deficiency (*n* = 8; 10.8%), progressive familial intrahepatic cholestasis (*n* = 13; 17.6%), congenital bile acid synthesis defects (*n* = 4; 5.4%), Dubin-Johnson syndrome (*n* = 3; 5.4%), cystic fibrosis (*n* = 1; 2.7%), and idiopathic cholestatic liver disease (*n* = 2; 2.7%).

**Table 1:** Baseline clinical characteristics of the study participants (*n* = 74)

**Characteristics**	**Value**
**Male gender of children, n(%)**	39 (52.7%)
**Age of children, n(%)**	
6–8 months	31 (41.9%)
9–11 months	16 (21.6%)
12–24 months	27 (36.5%)
**Household monthly income per capita, n(%)**	
<5000 RMB	50 (67.6%)
>5000 RMB	24 (32.4%)
**Literacy of primary care provider**, **n(%)**	
High school or below	44 (59.5%)
College degree or above	30 (40.5%)
**Presence of cholestasis, n(%)**	55 (74.3%)
**Total bilirubin (**μmol**/L),** median (IQR)	65.1 (19.0–183.8)
**Direct bilirubin (μmol/L),** median (IQR)	48.9 (12.3–112.7)
**GGT (IU/L),** median (IQR)	87.0 (34.0–315.0)
**ALT (IU/L),** median (IQR)	110.0 (67.8–211.5)
**AST (IU/L),** median (IQR)	137.0 (79.5–248.0)
**25-hydroxy-vitamin D (ng/ml),** median (IQR)	12.8 (8.6–24.6)

Abbreviations: *GGT* Glutamyl transpeptidase, *ALT* Alanine aminotransferase, *AST* Aspartate aminotransferase

### Feeding practice

Only 16.2% (12/74) of the children had appropriate feeding practices, and the median ICFI score was 6, with a range of 2–18.

#### Diary product intake

The intake rates of dairy products were breastfeeding 25.7% (19/74), medium-chain triglyceride (MCT) formula feeding 39.2% (29/74), and regular formula feeding 44.6% (33/74).

#### Complementary feeding practices

The rate of complementary feeding was 68.8% (51/74), and the mean age of adding complementary food was 6.9 (SD 1.7) months. For children consuming complementary foods, their dietary diversity involved single food groups with low energy density. A total of 66.7% (10/15) of children aged 6–8 months and 45.5% (5/11) of children aged 9–11 months had carbohydrates as a single source of complementary foods and had no intake of animal meat (meat, fish, poultry and liver/organ meats), eggs or bean products, while in the age group 12–24 months, 52.0% (13/25) of children had eggs less than 2 days/week, 60.0% (15/25) had meat less than 2 days/week, and 60.0% (15/25) had no intake of bean products (Table 2).

**Table 2:** Infant and Young Child feeding index (ICFI) components by age group

**Components**	**Age group**					
	**6–8 months (*n*** **= 31)**		**9–11 months (*n*** **= 16)**		**12–24 months (*n*** **= 27)**	
	**classification**	**n (%)**	**classification**	**n (%)**	**classification**	**n (%)**
**Dietary diversity**	0 group	16 (51.6%)	0–1 group	7 (43.8%)	0–2 groups	8 (29.6%)
	1–2 groups	12 (38.7%)	2–3 groups	4 (25.0%)	3–4 groups	12 (44.5%)
	> 3 groups	3 (9.7%)	> 4 groups	5 (31.2%)	> 5 groups	7 (25.9%)
**Meal frequency**	0 meal	16 (51.6%)	0–1 meal	7 (43.8%)	0–2 meals	11 (40.7%)
	1 meal	8 (25.8%)	2 meals	7 (43.8%)	3 meals	16 (59.3%)
	> 2 meals	7 (22.6%)	> 3 meals	2 (12.4%)	> 4 meals	0 (0.0%)
**Egg frequency**	0 day	27 (87.0%)	0 day	11 (68.8%)	0–2 days	15 (55.6%)
	1–2 days	2 (6.5%)	1–3 days	3 (18.8%)	3–4 days	6 (22.2%)
	> 3 days	2 (6.5%)	> 4 days	2 (12.5%)	> 5 days	6 (22.2%)
**Meat frequency**	0 day	29 (93.6%)	0 day	11 (68.8%)	0–2 days	17 (63.0%)
	1–2 days	1 (3.2%)	1–3 days	4 (25.0%)	3–4 days	5 (18.5%)
	> 3 days	1 (3.2%)	> 4 days	1 (6.2%)	> 5 days	5 (18.5%)
**Fruit frequency**	0–1 day	26 (83.9%)	0–2 days	9 (56.3%)	0–3 days	12 (44.4%)
	2–3 days	4 12.9%)	3–4 days	2 (12.5%)	4–5 days	4 (14.8%)
	> 4 days	1 (3.2%)	> 5 days	5 (31.2%)	> 6 days	11 (40.8%)
**Grain frequency**	0–4 days	20 (64.5%)	0–4 days	4 (25.0%)	0–5 days	1 (3.7%)
	> 5 days	11 (35.5%)	> 5 days	12 (75.0%)	> 6 days	26 (96.3%)

#### Medium-chain triglyceride intake

The rate of MCT intake was 40.5% (30/74), and among 30 children, 29 received MCTs entirely from specialized formula, while only one child was fed with MCT via the complementary foods.

### Determinants of inappropriate feeding practices

The bivariate analysis showed that the rate of inappropriate feeding practices was potentially associated with the age of the child and the literacy of the primary care provider. After controlling for potential confounding factors, the literacy of the primary care provider was significantly related to inappropriate feeding practices (Table 3). Parents without a college degree had a higher risk of inappropriate feeding behaviors than college-educated parents (adjusted OR 5.52; 95% CI 1.29, 23.66).

**Table 3:** Determinants of inappropriate feeding practices

**Variables**	**Rate of inappropriate feeding practice**	**Adjusted OR** **(95% CI)**	***p*-Value**
**Year of child**			0.756
< 12 months	87.2%	1.24 (0.32–4.75)	
> 12 months	77.8%	1.00	
**Primary care provider’s literacy**			0.021
High school or below	93.2%	5.52 (1.29–23.66)	
College school or above	70.0%	1.00	

### Feeding experiences of parents

Ten mothers and one father of children participated in the interviews (Table 4). Three main themes emerged from the thematic analysis: (1) lack of knowledge of feeding; (2) misunderstanding of the feeding process; and (3) lack of importance of complementary foods. An overview of the themes, subthemes and related quotations of the parents is provided in Table 5.

**Table 4:** Characteristics of parent interviews

**Number**	**Age of child** **(months)**	**Gender of child**	**Literacy of interviewee**	**Monthly household income** **(RMB per capita)**
A	8	male	junior college	>5000
B	12	female	primary school or below	1000–2999
C	8.5	male	junior high school	1000–2999
D	14	female	senior high school	3000–5000
E	10	male	junior high school	3000–5000
F	9	male	undergraduate	1000–2999
G	7.5	female	junior college	3000–5000
H	8	male	undergraduate	>5000
I	8	male	primary school or below	3000–5000
J	19	female	postgraduate	>5000
K	13	male	senior high school	>5000

Abbreviations: *RMB* Renminbi

**Table 5:** Themes, sub-themes and parent quotations

**Themes**	**Sub-themes**	**Parent quotations**
Lack of knowledge of feeding		“I tried to search information on the internet, but I still didn’t know what should I feed him, to avoid making mistakes, I only fed my baby with milk.” (participant C) “Children with jaundice must be different from normal children, I was not sure which kind of food could benefit her and which kind of food would cause damage to her.” (participant G)
Misunderstanding of the feeding process	Animal source food and oil will cause damage to the liver	“My baby had poor liver function, I felt flesh foods and eggs would aggravate his condition therefore I did not dare to feed him with these foods.” (participant B) “I thought flesh foods should be limited from my baby’s diets, because these foods did no good to the liver.” (participant K)
	The need to control the intake due to the poor digestive function	“I felt these children with jaundice had poor ability to digest, so they shouldn’t be fed too much, especially eggs and flesh foods.” (participant H)
Lack of importance of complementary foods		“Milks seemed to contain more nutrients, so I did not feed my child complementary food until he aged 11 months.” (participant D)

#### Lack of knowledge of feeding

All parents expressed their confusion about feeding their children, especially concerning the varieties, quantity and the introduction age of complementary food. There seemed to be a lack of information provided by the healthcare system, and other information sources might be difficult to find on Chinese websites. “I am completely blank and tried to look for information on the internet, but there was little relevant information. I am really at a loss and don’t know how to feed him, and am afraid wrong food would damage his health” (participant A). The lack of information was confirmed by another mother, who said, “I felt guilty every time seeing my child was so thin, but I had no idea how to feed him to help him get fat” (participant E). The consequence of the absence of support to parents with regard to feeding practices is identified in the second theme.

#### Misunderstanding of the feeding process

All but one parent thought that their children should be treated as abnormal and their diets should be as mild as possible. This theme led to two subthemes. The first subtheme was ‘*Animal source food and oil will cause damage to the liver’,* where parents expressed their ideas and anxiety about using certain foods. One parent said, “I thought food such as eggs, meats, and oil should be limited from my child’s diet because these foods could cause damage to the liver. Other mothers in the communication group shared the same idea with me” (participant E). The parents’ ideas about using certain foods sometimes came from family and friends; these ideas were taken seriously by the parents and included statements such as, “People around me said the diet of children with poor liver function should be as bland as possible. Therefore, my child just had porridge, rice, and noodles besides milk” (participant G). This could lead to misconceptions such as those expressed by one parent, “I felt food like meat, eggs, and oil could burden the liver and aggravate the liver function” (participant J). The second subtheme was ‘*the need to control the intake due to the poor digestive function’*. For example, one parent expressed, “My mother-in-law always tried to feed baby the egg yolk, but I worried that my baby couldn’t ingest it and refused to feed these foods” (participant F).

#### Lack of importance of complementary food

Several parents thought that milk had higher nutritional value than complementary food and gave no importance to complementary food: “I had never tried to introduce complementary food because I thought milk contained higher nutritional value and worried my baby would be even thinner after decreasing the volume of milk” (participant C) and “I felt milk had more nutrients than complementary food and therefore fed her milk as much as possible” (participant G).

## Discussion

The aim of our study was to evaluate the feeding status of children with chronic cholestatic liver diseases and to identify their parents’ experiences of feeding practices. Overall, the feeding practices of children appeared to fall short of the recommendations, specifically those related to medium-chain triglyceride oil supplementation and complementary feeding. Lower literacy of the primary care provider was related to inappropriate feeding practices. The interviews with parents indicated that parents had limited feeding knowledge that influenced their daily feeding practices.

Children with chronic liver disease have a greater energy requirements than healthy children [23, 24], and a decrease in the oral intake could predispose these children to a worse nutritional status [25]. Children older than 6 months need to gain some energy and nutrients from complementary feeding, and parents’ nutritional knowledge and feeding practices have significant impact on children’s growth and development. According to the current guidelines for nutritional management, feeding practices should be maintained as close to those of healthy age-matched children as possible [7–9]. However, in our study, most children were not introduced to complementary foods in a reasonable manner. The consumption of food from animal sources was suboptimal, and carbohydrates were the main source of energy in the complementary foods, with a lack of adequate amounts of meat and eggs. Inappropriate feeding behaviors resulted in a low energy density of the meals that did not contribute to the child’s growth and development.

These feeding practices did not follow the recommendations of the current guidelines, which recommend that the total energy intake should be 100 to 150% of that for an ideal weight, guided by the severity of failure to thrive, and that 3–4 g/kg/d of protein is necessary for children without a risk of hepatic encephalopathy [7–9]. However, these results were not in line with those of a study performed in Japan, which showed that 20% of children with Alagille syndrome had low dietary intake [10]. The variation among these studies could be due to sociocultural differences and different definitions of inadequate feeding. The Japanese study was conducted before the publication of feeding guidelines for children with chronic cholestasis, and inadequate intake was defined as consuming < 2/3 of the RDA. In our study, we defined inadequate intake as ICFI < 14 according to current guidelines.

Another important finding of our study was that the majority of parents were unaware of children’s specific nutritional requirements for MCTs. MCTs are absorbed directly through the portal venous system without the emulsification with bile [26], and supplementing MCTs can prominently decrease steatorrhea and improve growth in children with cholestasis [27, 28]. Thus, the 30–50% of total lipids are recommended to be given as MCTs in cholestatic patients, and in older children, MCT oil can be added to meals to increase energy density [8]. However, in our study, only a few children had enough intake of MCTs, and the source of MCTs was almost exclusively from specialized MCT formula. Only one mother reported that she added MCT oil in the complementary food, but she gave a small amount because of unpalatability. Compared with MCT oil, coconut oil may be a preferable choice, because 60–70% of the components in coconut oil are MCTs [29] and coconut oil does not make the food unpalatable [8, 20].

The deficiency of fat-soluble vitamins (A, D, E, and K) among children with chronic cholestasis are prevalent, which are primarily caused by fat malabsorption. Increasing oral fat intake may increase the total quantity of fat absorbed and improve growth and development despite causing steatorrhea [8]. However, in this study, the total fat intake was insufficient due to the inadequate intake of animal foods and vegetable oil. Future studies should further determine associations between the dietary intake and level of serum fat-soluble vitamins.

A logistic regression model was used to further evaluate the predictors of inappropriate feeding practices. The results indicated that after adjustment for covariates, only the literacy of the primary care provider was an independent predictor of inappropriate feeding practices. However, household monthly per capita income, a predictor of feeding behaviors among normal children [30, 31], was not significantly associated with feeding practices in children with chronic cholestatic liver diseases in this study. This finding was consistent with clinical observations; due to the low morbidity of the disease and the absence of reliable information sources, parents generally lacked science-based feeding knowledge. It was not the economic factor but incorrect ideas that contributed to inappropriate feeding practices.

Appropriate feeding of infants and young children feeding is required for optimal childhood nutrition, health and development [32]. To further explore the barriers of parents’ appropriate feeding behaviors and to provide practical information for clinical nutritional interventions to promote behavioral changes, we conducted interviews with parents. The findings indicated that a lack of knowledge of feeding, misunderstanding of the feeding process, and lack of importance of complementary foods emerged as important barriers to appropriate feeding practices. The most common misunderstanding to emerge was that animal source foods and oil will cause damage to the liver. One study in the Democratic Republic of the Congo also showed that the main barriers to optimal infant and young children feeding practices included a lack of knowledge and the presence of misconceptions among parents [33]. Another study on feeding practices in the Bolivian Andes found that parents’ lack of understanding of the importance of complementary feeding contributed to inappropriate feeding behaviors [34]. Moreover, due to the absence of effective interventions from clinical professionals, these wrong concepts and behaviors of parents spread among the community and became cumulatively reinforced, leading to a widespread misunderstanding about feeding practices.

Our study warrants some limitations. Firstly, this was a single-center study and our findings therefore may not be generalized. However, Children’s Hospital of Fudan University has been authorized by the government to be the first center of pediatric liver diseases in China. Children from all over the nation are admitted and treated in this hospital, thus for the Chinese region, our sample can be considered representative to some extent. Secondly, we did not assess children’s nutrients intake through dietary records. However, ICFI is a comprehensive index and has been widely used to assess dietary intake of children aged 6–24 months in China. Therefore our study provided important information for clinical practices.

The clinical implications of our study are related to healthcare professionals. Without access of parents to reliable sources of feeding knowledge, healthcare professionals play an important role in influencing parents to adopt positive practices. It is critical to establish a multidisciplinary care team to implement nutritional management targeting social and cultural family circumstances. These management strategies increase parents’ access to scientific knowledge about nutrition and the special nutritional needs of children so that parents can optimize their feeding behaviors and improve the nutritional status of children. Notably, considering that parents had serious misunderstanding about how to feed children with chronic cholestasis, appropriate interventions, such as providing various education materials for parents, should therefore be included in clinical disease management to enhance compliance with the nutritional plan.

## Conclusions

Nutrition received during the first 2 years not only influences children’s physical development, but also profoundly affects neurobehavior development and healthy dietary habits. As healthcare providers, we do not know enough about how children with chronic cholestatic liver diseases are fed, what hinders appropriate feeding practices, and what makes further interventions work. This study adds contextual understanding to the feeding practices in children with chronic liver diseases by adopting a mixed-method study. The results showed that feeding practices in these children are characterized by a lack of dietary diversity and an inadequate intake of MCTs, and feeding practices may be influenced by parents’ lack of feeding knowledge, their misunderstanding of the feeding process, and their lack of importance of complementary food. We hope that the findings in this study will provide practical information for clinical nutritional interventions to make impactful, cost-effective, targeted and sustainable interventions, so as to promote parents’ behavioral changes among parents, improve children’s growth and prevent stunted growth.

## Supplementary information

**Additional file 1.** The scoring system of the infant and child feeding index for children aged 6–24 months.

## Data Availability

The datasets used and analysed during the current study are available from the corresponding author on reasonable request.

## Abbreviations

RMB: Renminbi
GGT: Glutamyl transpeptidase
ALT: Alanine aminotransferase
AST: Aspartate aminotransferase
