# Supplementary material for: Feeding practices in 6–24-month-old children with chronic cholestatic liver diseases: a mixed-method study
Source: BMC Pediatr. 2020 Aug 24;20:395. doi: 10.1186/s12887-020-02290-8 (PMC7445932; doi:10.1186/s12887-020-02290-8)
Supplement: Supplementary file 1 — Additional file 1. The scoring system of the infant and child feeding index for children aged 6–24 months. [file 12887_2020_2290_MOESM1_ESM.doc]

**Supplemental Content 1:**The scoring system of the infant and child feeding index for children aged 6-24 months.

| **Variables** | **6-8 months** | **9-11 months** | **12-24months** |
| --- | --- | --- | --- |
| **Breastfeeding** | No = 0; Yes = 2 | No = 0; Yes = 2 | No = 0; Yes = 1 |
| **Bottle feeding** | No = 1; Yes =0 | No = 1; Yes =0 | No = 1; Yes =0 |
| **Dietary diversity**  **(past 24h)** | 0 type = 0  1-2 types = 1  >3 types = 2 | 0-1 type = 0  2-3 types = 1  >4 types = 2 | 0-2 types = 0  3-4 types = 1  >5 types = 2 |
| **Frequency of food for the past 7days** | | | |
| grains | 0-4 days/week=0  >5 days/week=1 | 0-4days/week=0  >5 days/week=1 | 0-5 days/week=0  >6 days/week=1 |
| vegetables | 0-1 day/week=0  2-3 days/week=1  >4 days/week=2 | 0-2 days/week=0  3-4 days/week=1  >5 days/week=2 | 0-3 days/week=0  4-5 days/week=1  >6 days/week=2 |
| fruits | 0-1 day/week=0  2-3 days/week=1  >4 days/week=2 | 0-2 days/week=0  3-4 days/week=1  >5 days/week=2 | 0-3 days/week=0  4-5 days/week=1  >6 days/week=2 |
| eggs | 0 day/week=0  1-2 days/week=1  >3 days/week=2 | 0 day/week=0  1-3 days/week=1  >4 days/week=2 | 0-2 days/week=0  3-4 days/week=1  >5 days/week=2 |
| flesh foods | 0 day/week=0  1-2 days/week=1  >3 days/week=2 | 0 day/week=0  1-3 days/week=1  >4 days/week=2 | 0-2 days/week=0  3-4 days/week=1  >5 days/week=2 |
| bean products | 0 day/week=0  1 day/week=1  >2 days/week=2 | 0 day/week=0  1 day/week=1  >2 days/week=2 | 0 day/week=0  1 day/week=1  >2 days/week=2 |
| dairy products | 0-3 days/week=0  >4 days/week=1 | 0-4 days/week=0  >5 days/week=1 | 0-3 days/week=0  4-5 days/week=1  >6 days/week=2 |
| **First formula milk feeding time** | <4 months or >9 months = 0; 4-5 months = 1; 6-8 months = 2 | | |
| **First complementary feeding time** | <4 months or >9 months = 0; 4-5 months = 1; 6-8 months = 2 | | |
| **Meal frequency** | 0 meal/day=0  1meal/day=1  >2 meals/day=2 | 0-1meal/day=0  2-3meals/day=1  >4 meals/day=2 | 0-2 meals/day=0  3 meals/day=1  >4 meals/day=2 |
| **Total scores** | 23 | 23 | 23 |
| **Appropriate feeding practice** | >14 | >14 | >14 |
